# Effects of Beech Wood Surface Treatment with Polyethylenimine Solution Prior to Finishing with Water-Based Coating

**DOI:** 10.3390/polym17010077

**Published:** 2024-12-30

**Authors:** Tanja Palija, Milica Rančić, Daniela Djikanović, Ksenija Radotić, Marko Petrič, Matjaž Pavlič, Milan Jaić

**Affiliations:** 1 Faculty of Forestry, University of Belgrade, 11030 Belgrade, Serbia; milica.rancic@sfb.bg.ac.rs (M.R.); milan.jaic@sfb.bg.ac.rs (M.J.); 2 Institute for Multidisciplinary Research, University of Belgrade, 11108 Belgrade, Serbia; danielle@imsi.rs (D.D.); xenia@imsi.rs (K.R.); 3 Biotechnical Faculty, University of Ljubljana, 1000 Ljubljana, Slovenia; marko.petric@bf.uni-lj.si (M.P.); matjaz.pavlic@bf.uni-lj.si (M.P.)

**Keywords:** wood, polyelectrolyte, polyethyleneimine, water-based coating, wood-coating interface, wood surface finishing

## Abstract

The surfaces of beech wood samples were treated with polyethylenimine (PEI) solutions at three different concentrations—0.5%, 1% and 2%—and two molecular weights—low molecular weight (LMW) and high molecular weight (HMW). The effects of PEI surface treatment of wood were characterized by FT-IR spectroscopy, the penetration depth of PEI (EPI fluorescence spectroscopy), the bonding position of PEI (by SEM), the wetting and surface energy, and the water uptake. After PEI treatment, the samples were coated with a water-based transparent acrylic coating (WTAC). The dry film thickness, the penetration depth of the coating, the adhesion strength and the surface roughness of the coated wood surface were evaluated. EPI fluorescence and SEM micrographs showed that PEI HMW chains were deposited on the surface, in contrast to PEI LMW, which penetrates deeper into layers of the wood cells. Treatment with a 1% PEI HMW solution resulted in a 72% reduction in water uptake of the wood (compared to untreated samples after 5 min of applying water droplets to the surface) and a 23.2% reduction in surface energy (compared to untreated samples) while maintaining the adhesion strength of the applied WTAC. The lower water uptake of the treated wood samples reduced the roughness of the coated surface, which is particularly important when the wood surface is finished with water-based coatings.

## 1. Introduction

Wood as a carbon-neutral natural material and innovative wood products have attracted great interest in recent years due to increasing environmental awareness. However, in order to extend the service life of wood products, it is necessary to protect their surfaces from environmental influences. Surface protection is usually achieved by coating. The main disadvantage of conventional coatings based on organic solvents is the emission of volatile organic compounds (VOCs). In recent years, the coatings industry has faced increasing pressure to reduce its overall environmental impact [1]. For this reason, environmentally friendly materials for the surface protection of wood, such as water-based coating formulations, have a growing share of the coatings market. In the wood industry, the use of water-based coatings has increased significantly in recent decades [1]. Today, water-based resins for coating formulations are in many ways equivalent to or better than their solvent-based counterparts [2]. Due to lower VOC exposure, water-based coatings are less toxic and flammable and easy to clean [3]; they have higher water vapor permeability and water uptake than their solvent-based counterparts [4]. New trends in water-based coatings are mostly based on further reducing greenhouse gas emissions by using bio-based materials that are sustainable both in terms of the production process and the raw materials used [5]. Given that wood is a hygroscopic material, the main disadvantage of using water-based coatings is that it swells upon contact with water, which manifests itself in a raising of the wood fibers and an increase in the roughness of the coated surface [6,7]. In addition, the swelling of the wood leads to a narrowing of the coating’s path of movement into the interior of the wood tissue, which can affect the depth of penetration, which is a prerequisite for achieving adequate adhesion between the coating and the wood substrate [8].

Previous studies have shown that it is possible to utilize the electrical potential of wood, which is due to the dissociation of functional groups of the basic polymeric structures, especially the carboxyl groups [9], for the electrostatic binding of various materials to the wood surface. Polyelectrolytes (PEs) are charged macromolecules with a positive or negative net charge depending on the pH value of the environment. Most of them are water-soluble [10] and can dissociate in polar solvents, such as water, producing counterions that are dispersed in the solution. PE coatings can change the charge, surface energy and mechanical properties of various substrates. Furthermore, functionalized PE coatings are considered a potential solution in the context of green chemistry [11]. Since cellulose, like wood, has a negative electrical surface potential in moderately acidic or neutral environments, cationic polyelectrolytes can be used to bind efficiently to negatively charged wood surfaces. In addition, the water from the polyelectrolyte solution has a positive effect on increasing the electrical conductivity of the wood (a 10^10^ to 10^13^ fold increase in electrical conductivity when the moisture content of the wood increases from near zero to fiber saturation, according to Simpson and TenWolde (2007) in [12]).

In the previous study, the influence of the application of commercially available cationic polyelectrolyte solutions (twelve different types) to samples of oak (*Quercus robur*) and Scots pine (*Pinus sylvestris*) on the extent of water uptake was investigated [13]. The adsorbed polyelectrolytes hindered the penetration of water into the pore system of the wood, according to the results of the relative water uptake, which indicates the percentage ratio of water uptake to total water consumption. However, with the different polyelectrolyte solutions, the oak and pine samples showed a different degree of decrease in water uptake. Compared to untreated oak and pine samples, treatment with a 1% PEI solution reduced the relative water uptake of the treated samples by 41.1% and 36.9%, respectively. The concentration and molecular weight of the aqueous polyelectrolyte solutions proved to be key in maximizing the modification effect. In the study investigating the degree of adsorption of poly(AM-co-DADMAC) (copolymers of acrylamide and diallyldimethylammonium chloride) in the cell wall of oxygen-bleached, never-dried kraft pulp, only polyelectrolytes with a charge density and a molecular weight below a critical value could be adsorbed in the cellulose fiber cell wall [14]. When investigating the influence of the molecular weight of poly(AM-co-DADMAC) (8 × 10^3^ g/mol; 1.1 × 10^5^ g/mol; 4.0 × 10^5^ g/mol) on the adsorption of cellulose fibers, the polyelectrolyte with the lowest molecular weight showed a significantly higher degree of adsorption due to its penetration into the cell walls. In another study [15], it was found that pretreatment of beech wood with a 1% high-molecular-weight PEI solution resulted in a reduction in the roughness of the wood coated with a water-based coating, especially when the initial roughness was higher. This result shows that when using water-based coatings for the surface treatment of wood, surface treatment with a polyelectrolyte solution can effectively replace more intensive sanding (in terms of the number of sanding steps and grit) to produce a coated surface with lower roughness. In addition, the PEI surface treatment had no effect on the adhesion strength of the water-based acrylic coating on the treated samples. In testing the adhesion strength of three different types of coatings for exterior application on redwood, PEI and aminosilanes were used for chemical bonding of the coating to the substrate by the grafted molecular brushes [16]. The highest adhesion strength of the coatings (measured by visual assessment of the delamination effect using the cross-cut method) was observed after the aforementioned treatment, which was preceded by surface oxidation with corona discharge and/or oxidation.

PEI is considered an environmentally friendly substance that is safe and easy to use [17]. It is used as a component in the production of environmentally friendly adhesive without formaldehyde emissions [18,19], as well as for the formation of a coating on the wood surface through a layer-by-layer system [20] and polyelectrolyte complexes [21] to achieve flame-retardant wood. One of the main advantages of PEI is the ability to form hydrogen bonds [17], which is due to the large number of amino groups, whereby each nitrogen-bonded hydrogen atom within the amino group can be involved in the formation of hydrogen bonds.

The optimal parameters of the PEI solution for surface treatment must be determined in relation to the properties of the final product, i.e., the wood coated with a water-based coating. However, it is not known how the ability of PEI to bind water affects the behavior of water-based coatings that come into contact with the treated wood surface in terms of wetting, penetration and bonding. In this context, it is important to know the effects of PEI absorption on chemical, structural and physical changes in wood surfaces. In order to exploit the potential of PEI in the field of wood finishing with water-based coatings, it is important to understand the indirect effects of the parameters of the polyelectrolyte solution on the properties of the finished wood.

Therefore, the main objective of this study is to investigate the possible effects of using the weak polycation PEI as a pretreatment in the finishing of wood surfaces with water-based coatings. The effects of different concentrations and molecular weights of PEI solutions on the properties of the treated wood surface were investigated. It was then analyzed how the pretreatment of wood with PEI affects the key properties of the coated wood when using water-based coatings. The low-molecular-weight PEI and the high-molecular-weight PEI were used to modify the penetration effects of the polyelectrolytes into the wood surface.

## 2. Materials and Methods

### 2.1. Preparation of Wood Samples

Samples of beech wood (*Fagus moesiaca* C.) with dimensions of 225 mm × 30 mm × 10 mm were obtained by cutting from boards and then conditioned for two months in a climate chamber with controlled environmental parameters (t = 15 ± 1 °C and φ = 50 ± 2%). All samples were prepared from quarter-sawn boards (radial cut) to exclude the influence of the anatomical direction on the test results, taking into account its influence on the differences in swelling of the wood in contact with water. After the conditioning phase, the average wood moisture content was 9.53% (measured according to ISO 13061-1:2014), which corresponds to the percentage of moisture content of the wood in wood end-use products. After conditioning, the samples were sanded on a wide-belt sanding machine (LSM 8, Heesemann, Germany) in a four-stage system with the following sanding belt grits: P60, P80, P100 and P150. The sanding speed was 20 m/s and the conveyor belt speed was 16.5 m/min. Before applying the polyelectrolyte solution, the surface of the samples was sanded manually using a hand sander and the abrasive belt grit size P150 to obtain a fresh surface. Smaller test samples were prepared with the dimensions: 90 mm × 30 mm × 10 mm; 50 mm × 30 mm × 10 mm and 30 mm × 30 mm × 10 mm, for various subsequent tests.

Aqueous PEI solutions of low and high molecular weight with similar pH values were used for the surface treatment of the samples. The specifications of the polyelectrolytes used are listed in Table 1.

Deionized water with an electrical conductivity of approx. 0 µS/cm was used to prepare the PEI solutions. The diluted solutions were homogenized with a magnetic stirrer for 2 h at a speed of 1000 rpm. The chosen concentrations are consistent with previous tests on cellulose fibers and veneer surfaces [9,14]. The polyelectrolyte solutions were manually applied to the surface of the samples using a sponge-coated roller.

After drying the samples under controlled ambient conditions (t = 23 ± 2 °C and φ = 50 ± 5%), WTAC (“AQUAL—basecoat for furniture”, Pitura, Belgrade, Serbia) was applied to the surface using a manual applicator with a fixed gap height at a theoretical wet film thickness of 200 µm. The basic properties of the applied liquid coating were tested and are shown in Table 2.

### 2.2. Test Methods for Wood Treated with PEI Solution

#### 2.2.1. Determination of the Chemical Characteristics of the Wood Surface After Treatment with Polyelectrolyte Solution

The changes in the chemical properties of the wood surface were investigated using FT-IR spectroscopy (MB-102 FTIR, BOMEM Michelfan, Frankfurt, Germany spectrophotometer in transmission mode). FT-IR images of untreated and PEI solution-treated wood samples were obtained by homogeneously mixing a small amount of finely dispersed wood flour with potassium bromide (KBr) and pressing it into a thin round plate. The wood powder that was used to form the pastille was obtained by sanding the surface layer of untreated and treated samples.

#### 2.2.2. Determination of Penetration Depth of PEI into Wood

**Figure 1:**
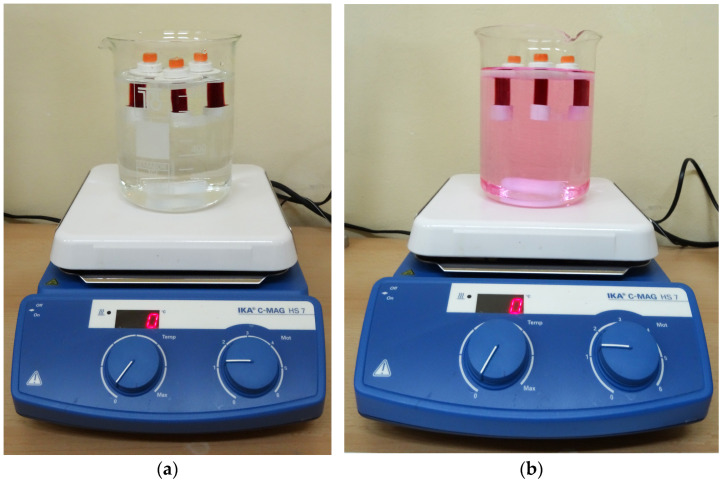
Dialysis of PEI LMW in deionized water: (**a**) at the beginning of dialysis; (**b**) 24 h after the start of dialysis.

The position of the polyelectrolyte in the wood tissue after application was determined with an EPI fluorescence microscope (Axio Observer Z1, Carl Zeiss Microscopy, Jena, Germany) by staining the polyelectrolyte with a fluorescent agent. Rhodamine B (Centrohem, Stara Pazova, Serbia) was used as a fluorescent staining agent due to its ability to form covalent bonds with PEI molecules. Staining of the polyelectrolytes with a fluorescent dye was performed according to a procedure similar to that described in the literature [27]. In total, 0.042 g of rhodamine B was dissolved in 1 mL of deionized water; then, 0.035 g of PEI LMW dissolved in 3 mL of deionized water was added to the solution. In this way, a solution with a concentration of rhodamine five times higher than that of PEI was obtained. The pH of the solution was adjusted to 9 by adding hydrochloric acid solution. The solution was stirred for 24 h with a magnetic stirrer. Excess rhodamine B was removed by dialysis using a dialysis device (Float-a-Lyzer, Spectrapor, Massachusetts, CA, USA) with a dialysis membrane whose pore size corresponds to the MWCO (Molecular Weight Cut Off) of the polyelectrolyte of 500–1000 Da for two weeks, with deionized water being exchanged every 24 h as a buffer (Figure 1). During the dialysis process, the buffer solution changes color due to the release of excess unbound dye. Dialysis of PEI HMW was performed using dialysis cassettes (Slide-a-LyzerTM, Thermo Scientific, Waltham, MA, USA) with a MWCO pore size of 10 kDa. Due to the larger difference in the size of the membrane aperture and the size of the molecules of the samples, PEI HMW dialysis was performed for 48 h, with a buffer change after 4 h, 8 h, and 16 h.

After completion of dialysis, aqueous solutions of polyelectrolytes were prepared at the desired concentrations and applied to wood samples measuring 50 mm × 30 mm × 10 mm. After drying, the surface of the samples was covered with self-adhesive tape and then coated with WTAC to prevent damage to the surface of the treated samples′ during preparation of the microtome samples. For better visual observation of the polyelectrolytes in the wood tissue cells, three filters were used to image the samples: 49 DAPI (excitation at 358 nm; emission at 461 nm), FAM (excitation at 490 nm; emission at 520 nm) and DsRED (excitation at 563 nm; emission at 581 nm). The application of the DAPI filter leads to autofluorescence of the wood cell wall, which is primarily due to the fluorescence of lignin as the main fluorescent component [28], which occurs at around 445 nm with an excitation of 360 nm [29]. The individual images taken with these three filters were combined into a single photo (using Photoshop). Finally, photos were taken in RBG mode with the red color assigned to the DsRED filter, the blue color to the FAM filter, and the green color to the DAPI filter.

#### 2.2.3. Determination of the Binding Position of Polyelectrolytes in the Wood Structure After Adsorption

A scanning electron microscope (SEM) (Tescan Vega TS 5130MM, Brno, Czech Republic) equipped with a back-scattered electron (BSE) detector was used to qualitatively evaluate the penetration depth of the polyelectrolytes. The SEM images were taken on representatives of PEI samples with a 1% concentration of different molecular weights (LMW and HMW).

The SEM images were taken on previously prepared microtome samples. The preparation of the samples for SEM analysis consisted of gluing the microtome samples to metal carriers with conductive double-sided carbon adhesive tape. The glued supports are coated with a very thin layer of gold (approx. 10–20 nm) by means of argon ionization under high voltage. The impact of argon ions on the cathode, i.e., the gold-coated chamber shell, causes gold atoms to break off and coat the sample. Due to the higher energy compared to secondary electrons, “backscattered” electrons can penetrate deeper into the surface of the sample. To investigate the effect of the polyelectrolyte treatment, an image was therefore generated by detecting bounced elastic electrons.

#### 2.2.4. Determination of Wetting Ability of Wood Surface Treated with PEI Solution

To assess the wettability of the treated surface of the samples with a water-based coating, the contact angle was measured using the drop method according to EN 828: 2009 (“sessile-drop test”) of water and WTAC droplets and then the surface energy of the wood was determined on the basis of the measured contact angles of liquids with known surface tension—water, formamide and diodomethane (according to Chibowski and Perea-Carpio (2002) in [30]). Liquid droplets with a volume of approx. 2 µL were dropped from a low height relative to the substrate surface. For each group, 5 test specimens (dimensions 90 mm × 30 mm × 10 mm) were selected on which four drops of the tested liquids were placed. The contact angle was measured in the direction of the wood fibers using the images taken with a video camera through an optical microscope (Olympus SZH, Olympus, Tokyo, Japan) with an objective (Olympus DF PLAN, Olympus, Tokyo, Japan) with a magnification of 1.5. The change in contact angle compared to the initial position, i.e., the zero position, was tracked for 25 s for water and water-based coating.

#### 2.2.5. Determination of Water Uptake of Wood Surface Treated with PEI Solution

The degree of water uptake was determined by placing water droplets on the surface of test samples measuring 30 mm × 30 mm × 10 mm. For each group of samples (control, treated with PEI LMW, and treated with PEI HMW), nine samples were used, and the narrower sides of the test tubes were coated with a coating to ensure water uptake only from the wide side of the samples. Five droplets of water (total approximate mass 0.2 g) were placed on each sample, using a syringe (TROJECTOR-3, TROGE, Hamburg, Germany) with an injection needle (0.4 mm × 19 mm). Excess water was removed from the surface of the sample with absorbent material. The degree of water uptake was expressed as a percentage of the increase in the mass of the sample after a certain time concerning the total mass of applied water droplets, according to the equation:

(1) $$a=\frac{m_{0}+m_{aw}}{m_{0}+m_{w}}\;\cdot \;100\;\left[\%\right]$$

where *a*—degree of water uptake; *m*_0_—the initial mass of the sample [g]; *m_aw_*—the mass of water absorbed by the sample [g]; *m_w_*—the mass of water applied to the surface of the samples [g].

Three test samples were used for each group to determine water intake at different time points: after 1 min, after 2, and after 5 min.

### 2.3. Methods of Testing the Properties of Dry Film Coating on the Surface of Wood Treated with PEI Solution

#### 2.3.1. Dry Film Thickness of Coating

**Figure 2:**
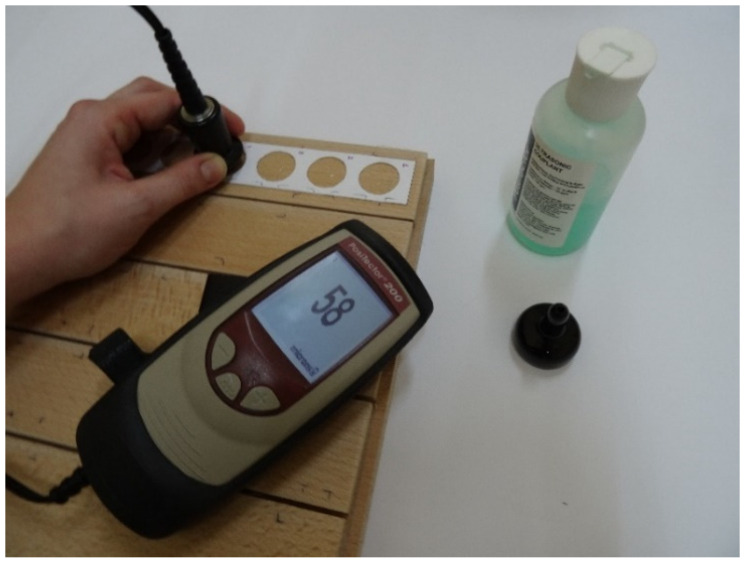
Dry film thickness measurement of water-based transparent acrylic coating.

The dry film thickness (DFT) of WTAC was measured using an ultrasonic gauge (according to EN ISO 2808: 2019 [31]). For each group of samples, the DFT was measured at 30 positions (Figure 2).

#### 2.3.2. Coating Penetration Depth

To determine the penetration depth of WTAC, the technique of superimposing images taken with an EPI fluorescence microscope with different filters was used. (The same method was used to determine the penetration depth of polyelectrolytes). The quantitative determination of the coating penetration depth was carried out with the computer program Image J (version 1.46). For each sample group, 30 microtome samples were prepared and photographed with EPI fluorescence microscopy. The total number of photomicrographs was 210.

**Figure 3:**
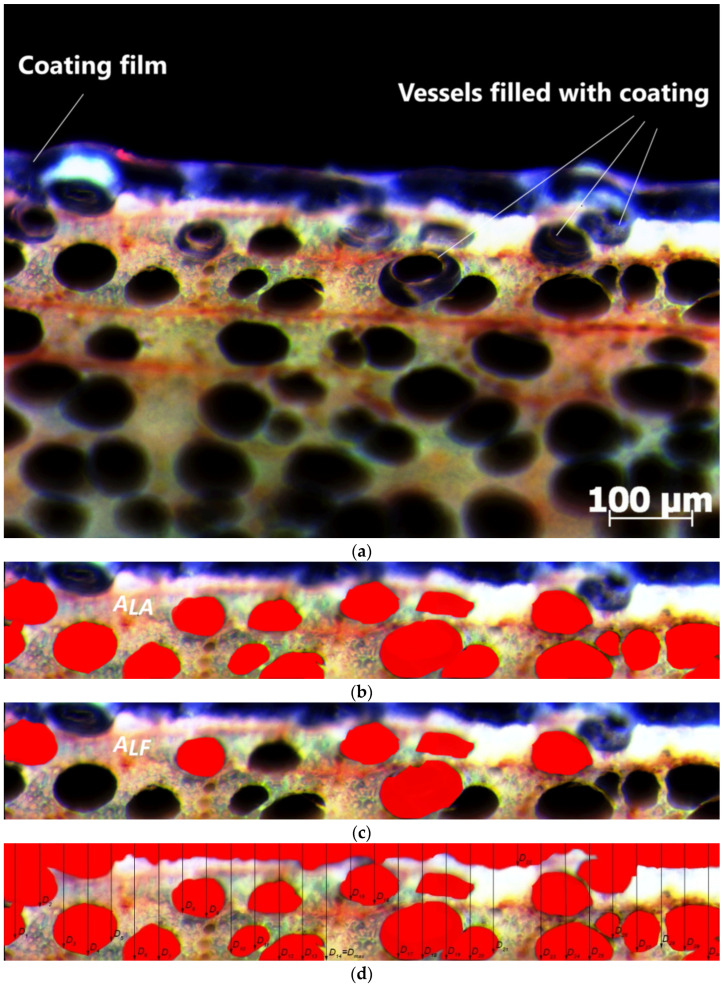
Calculation of coating penetration parameters: (**a**) snapshot of microtome sample; (**b**) area of available cell lumens (*A_LA_*) in the penetration surface (*PS*); (**c**) area of filled cell lumens (*A_LF_*) in the penetration surface (*PS*); (**d**) coating penetration depth at 30 positions and maximum coating penetration depth (*D_max_*) in the penetration surface (*PS*).

The maximum penetration depth of the coating (*D_max_*) and the average penetration depth of the coating (*D_av_*) were measured on each micrograph. For the calculation of the penetration depth parameters, the coating penetration surface (PS) is defined by the initial penetration plane passing through the highest point of the wood section (according to Van den Bulcke et al. (2010) in [32]) and the plane parallel to a previous plane passing through the lowest point where the coating is visible (Figure 3). Within the PS of each micrograph, the area of filled cell lumens (*A_LF_*) and the area of available cell lumens (*A_LA_*) were calculated. For the calculation of *D_av_*, each photomicrograph was divided into 30 sections of equal width for which the penetration depths were measured. *D_av_* was calculated as the mean of the 30 results for each photomicrograph.

For each recording, the filling of the available cell lumens (*LF*) with the coating within the coating penetration zone was calculated according to the formula:

(2) $$LF=\frac{A_{LF}}{A_{LA}}\;\cdot \;100\;\left[\%\right]$$

#### 2.3.3. Adhesion Strength of Coating

**Figure 4:**
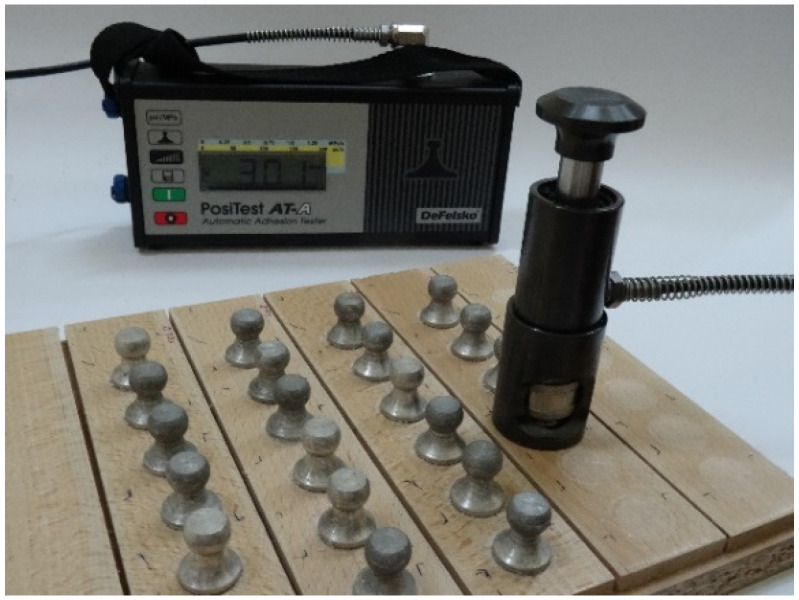
Determination of WTAC adhesion strength by the pull-off test.

The adhesive strength of WTAC was measured by the pull-off test according to EN ISO 4624: 2023 [33] using an automatic device with a hydraulic pump (POSITest AT-A, DeFelsko, Ogdensburg, NY, USA). Dollies with a diameter of 20 mm were glued to the coated surface of the samples using a 2K epoxy adhesive (Epoxy Universal, Bison, Zoetermeer, The Netherlands). The adhesive cured for 24 h under room climate conditions. After the adhesive had cured, the coating was cut around the circumference of the dollies which were then pulled in a vertical plane at a speed of 0.7 MPa/s until breakage (Figure 4). The adhesion strength of WTAC was measured for each sample group on 5 samples measuring 225 mm × 50 mm × 10 mm at six positions, i.e., a total of 210 measurements. The results of adhesion strength with the failure mode outside the adhesive coating plane were not considered in the statistical analysis.

#### 2.3.4. Roughness of the Coated Surface

**Figure 5:**
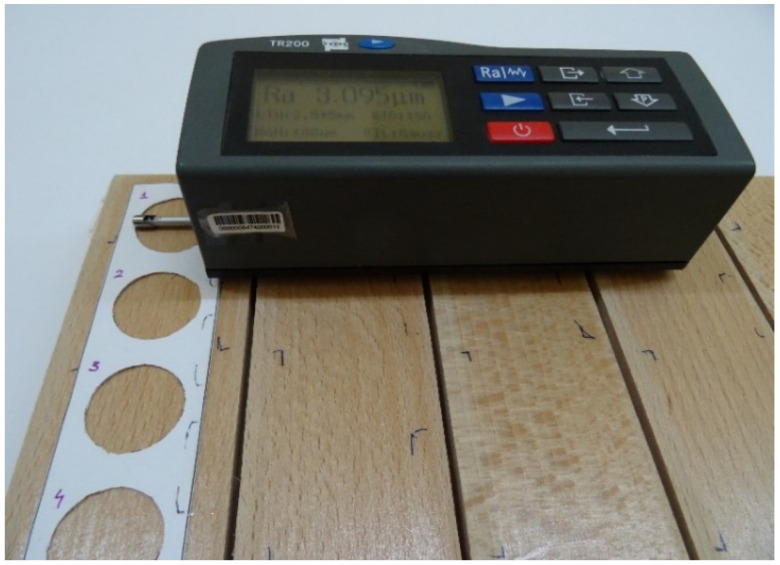
Measuring the roughness of the wood-coated surface.

A stylus contact tester (TimeSurf TR200, Beijing TIME High Technology Ltd., Beijing, China) was used to determine the surface roughness of coated samples (Figure 5). The device is equipped with a diamond-tipped needle (diameter 2 µm) that touches the surface with a force of 4 mN. The following surface roughness parameters were used to characterize the coated surface: *R_a_* (arithmetic mean deviation of the profile), *R_z_* (maximum profile height) and *R_t_* (total height of the profile) according to ISO 4287: 1997 [34], with a selected sampling length of 2.5 mm. The roughness was measured on samples with the dimensions of 225 mm × 50 mm × 10 mm at six positions on five samples, i.e., at a total of 30 measuring positions for each sample group.

### 2.4. Statistical Analysis

The results of the parameters DFT, adhesion strength, surface roughness and WTAC penetration dept were analyzed with SPPS IBM Statistics 20. These results were compared using analysis of variance (ANOVA) to determine whether there was a statistically significant difference between the result groups. The basis of ANOVA is the F-statistic, which determines the relationship between the variability within the variables (also known as error) and the variability between the variables being compared (also known as systematic variance). If the assumption of homogeneity of variances was met (by the Levine test), the ANOVA was performed. If the ANOVA revealed a statistically significant difference between the sample groups, a post hoc analysis was performed (Tukey’s HSD) to clarify between which groups significant differences were found. In cases where the assumption of homogeneity of variances was not met (different variances), the test results were checked using the Welch test. A post hoc test (Games–Howell) was used to determine which group differed from the others if the Welch test showed a statistically significant difference in the results between the groups. All tests were performed with a confidence level of 95% (*p* < 0.05).

## 3. Results and Discussion

### 3.1. FTIR Analysis

**Figure 6:**
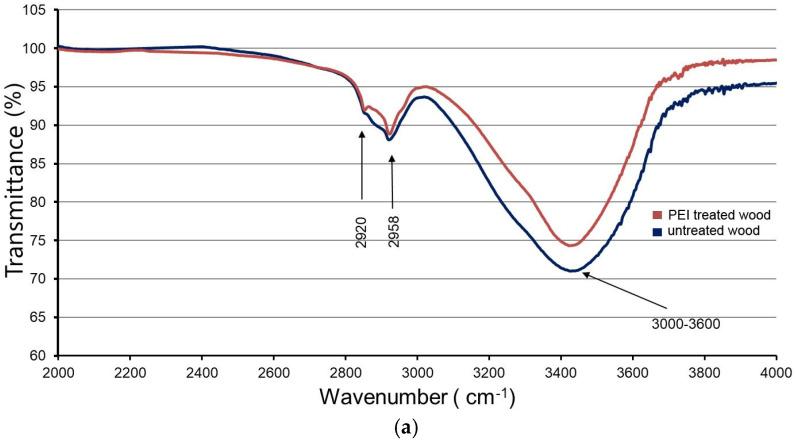

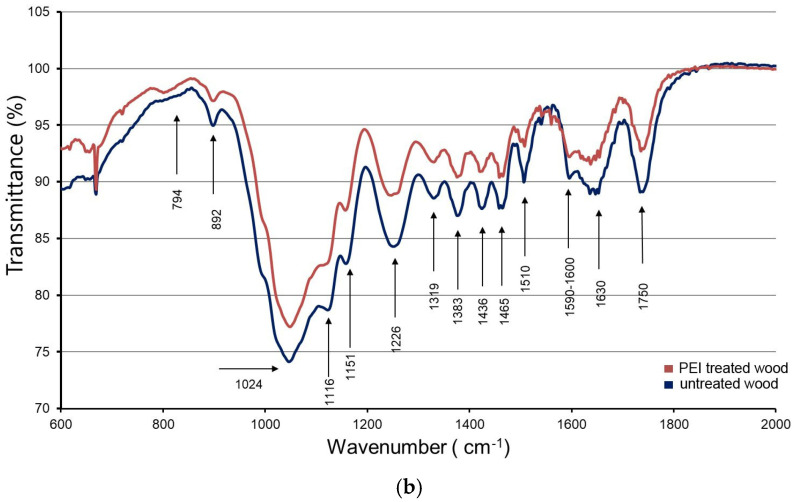
FT-IR spectra of untreated and samples treated with PEI solution in the range of 2000–4000 cm^−1^ (**a**) and from 600 to 2000 cm^−1^ (**b**).

FT-IR spectra show the presence of a broad absorption band in the range of 3600–3000 cm^−1^ (Figure 6a), which is attributed to the vibrations of hydroxyl groups (–OH) [21] and water adsorption [27]. In samples treated with polyelectrolyte solutions, the intensity of this band was reduced compared to untreated control samples. The decrease in intensity can be explained by the breaking of hydrogen bonds between the cellulose and hemicellulose chains during the penetration of PEI into the cell walls. Hydrogen bonds occur between neighboring cellulose chains as well as between hemicellulose molecules and cellulose chains in the amorphous region. According to Gibson [29], during the sorption and desorption process, water molecules move rapidly through the material from one free-binding site to another, followed by the formation of new hydrogen bonds and the breaking of old ones. During the swelling of wood, there is a spatial rearrangement of neighboring cellulose molecules, while during desorption, the tension created by the loss of water molecules reduces the distance between the newly formed regions. During water loss, the reduction in the distance between the cellulose and hemicellulose chains enables the formation of stronger hydrogen bonds between them.

In the samples treated with PEI solution, new hydrogen bonds are probably formed between the electronegative atom N and the protons of the wood cell wall components. In addition, hydrogen bonds can be formed between cellulose molecules in the amorphous region (intermolecular hydrogen bonds) and between parallel cellulose chains in the crystalline region (intramolecular hydrogen bonds). The vibration of the absorption band around 3335 cm^−1^ indicates intramolecular hydrogen bonds (according to Liang and Marchessault (1959) in [30]). The formation of hydrogen bonds between parallel cellulose chains in the crystalline region is possible due to the separation of the planes of parallel cellulose chains in an alkaline medium, whereby the crystal lattice is not disturbed but only transformed [32]. The high pH of the PEI solution (pH = 10) during treatment has an effect on the reorganization of the crystal structure and consequently on the swelling of the cell wall. 

The absorption bands at 2958 cm^−1^ and 2920 cm^−1^ show characteristic peaks, which are attributed to asymmetric and symmetric stretching vibrations of CH and CH_2_ groups, respectively [21]. A large number of clearly defined peaks can be observed in the 1800–1600 cm^−1^ range. The intensity of the vibrational band at 1750 cm^−1^, which is due to the stretching of the non-conjugated C=O bond in the carboxyl groups present in xylan [35], is lower in the samples treated with PEI solution, indicating a reduction in the content of carboxyl groups (Figure 6b). On the other hand, the appearance of a peak at about 1650–1580 cm^−1^ corresponds to NH bending in amide bonds (type II), indicating the chemical bonding of carboxyl groups of wood and charged nitrogen atoms of PEI. The formation of amide bonds was observed in the reaction of cellulose with PEI in a molar ratio of 4:1 [36].

The bands below 1600 cm^−1^ show no significant changes (Figure 6b) after treatment with PEI. The weak FT-IR band at 790 cm^−1^, which is attributed to the out-of-plane vibration of the NH ion, shows that PEI is only involved to a certain extent in the chemical bonding with the carboxyl groups of cellulose.

Analysis of the FT-IR spectra led to the conclusion that PEI molecules have an affinity for hydrogen bond formation due to the presence of nitrogen atoms, as well as the possibility of chemical bonding with the carboxyl groups of the wood. Consequently, there is not only the possibility of forming electrostatic interactions with oppositely charged atoms but also the ability to form bonds outside the electrostatic field.

### 3.2. Penetration Depth of PEI Solution

**Figure 7:**
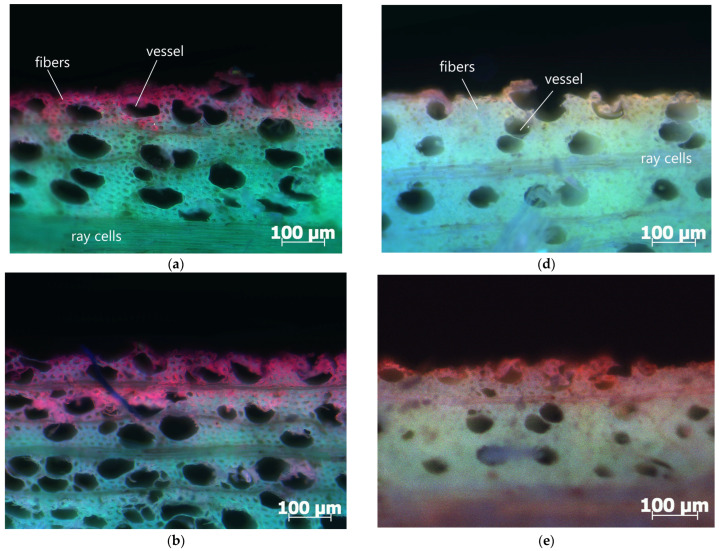

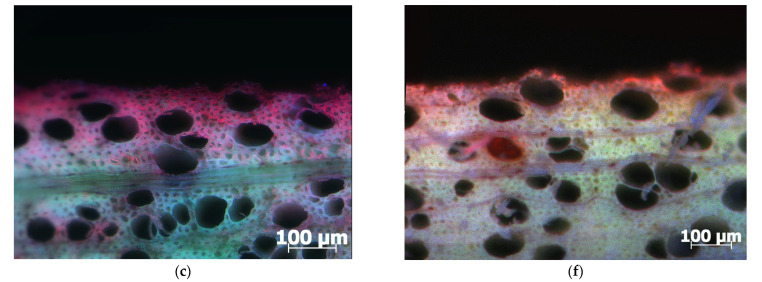
Images of penetration of low- (LMW) and high-molecular-weight (HMW) PEI solutions of different concentrations, stained with rhodamine B, into the surface layer of wood, obtained by overlaying images with DAPI, FAM, and DsRED filters from an epi-fluorescence microscope: (**a**) 0.5% PEI LMW; (**b**) 1% PEI LMW; (**c**) 2% PEI LMW; (**d**) 0.5% PEI HMW; (**e**) 1% PEI HMW; (**f**) 2% PEI HMW.

Figure 7 shows photographs of the penetration of PEI LMW and PEI HMW solutions of different concentrations obtained by computer overlay of images taken with three different filters (DAPI, FAM and DsRED). In the PEI-treated samples, a higher concentration of polyelectrolytes in the surface layers of the wood was observed in higher molecular weight samples (Figure 7d–f), compared to samples treated with a low-molecular-weight solution of this polyelectrolyte (Figure 7a–c). These results are consistent with previous studies on the influence of polymer molecular weight on penetration into the wood cell wall, in which it was found that only low-molecular-weight PEI (800 Da) could sufficiently penetrate the cell walls, while low- and high-molecular-weight PEI (800 Da and 750,000 Da, respectively) coated the lumens at high density [35]. It was observed (Figure 7) that the penetration depth of the PEI solution increased with increasing concentration regardless of the molecular weight. A lower penetration depth of the polyelectrolyte was observed in samples where the cell rays were located on the surface itself or in several rows of cells below the surface. This phenomenon can be explained by the transport paths of the matter through the wood tissue.

**Figure 8:**
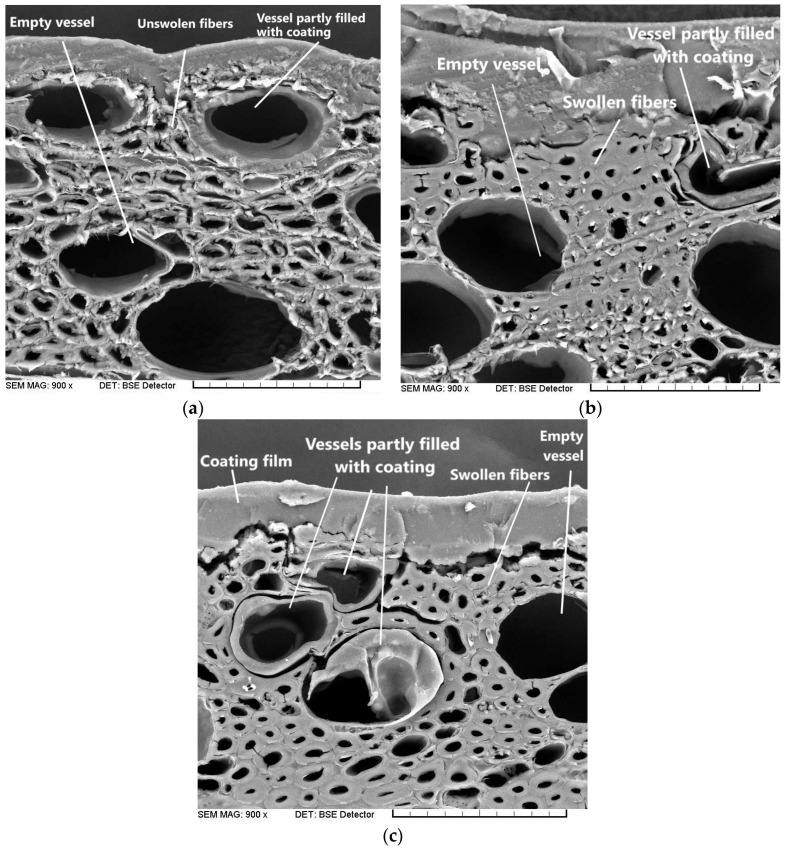
SEM images of wood samples protected by a coating layer (900× magnification): (**a**) untreated wood; (**b**) samples treated with a solution of 1% PEI LMW; (**c**) samples treated with a solution of 1% PEI HMW.

On the SEM image of untreated beech wood (Figure 8a), two main components can be distinguished: larger cells (vessels) with large lumens and thin walls and smaller cells (fibers) with thick walls. Fluid transport between the cells takes place through pits that connect neighboring individual cells. According to studies of the porosity of beech wood, the pore size of the fiber lumens is in the order of 0.5 to 2 µm [36]. The untreated samples (Figure 8a) and the samples treated with 1% PEI LMW or PEI HMW solutions (Figure 8b or Figure 8c) differ significantly in the thickness of their fiber walls. The change in the conformation of the cellulose molecules can be seen on a microscopic level by the swelling of the wood cell wall, which is confirmed by SEM images (Figure 8b,c). In addition, a “smoother texture” of the wood fibers can be seen in the surface layer of the treated wood samples. This phenomenon indicates that the cell walls were filled (bulking) with polyelectrolytes, which led to an increase in the volume of the walls themselves. The swelling of the fiber walls can be observed not only when the samples were treated with PEI LMW (Figure 8b), but also when they are treated with PEI HMW (Figure 8c). This result indicates that the swelling of the fibers is related to the water uptake from the aqueous PEI solution through hydrogen bonding with the hydroxyl groups of the wood cell wall polymers [37]. The samples treated with polyelectrolyte solutions (Figure 8b,c) showed a clear difference in the cell structure of the wood fibers in the form of thicker walls and narrower lumens in the surface layers of the wood compared to those in the deeper layers of the sample. This result confirms that PEI penetrates into the wood, as shown in the EPI images (Figure 7a–f).

Regarding the penetration of the WTAC coating into the beech wood samples, the filling of the vessels is only visible in individual cells of the first row of the untreated and treated samples. This result is consistent with other studies in which the penetration depth of water-based acrylic coatings into wood surfaces is relatively low compared to solvent-based coatings [38,39]. In the study on beech wood, the penetration depth of water-based acrylic coatings was limited to the surface layer of the wood cells (a few tens of micrometers); only individual lumens of larger vessels below the surface on the investigated surfaces were filled with coating [40]. The low penetration depth of the coating can be attributed to the high viscosity of the liquid coating due to the application technique used, which affects the spreading rate and uniformity of the applied coating on the surface [41].

### 3.3. Characterization of the Surface of the Treated Samples

**Figure 9:**
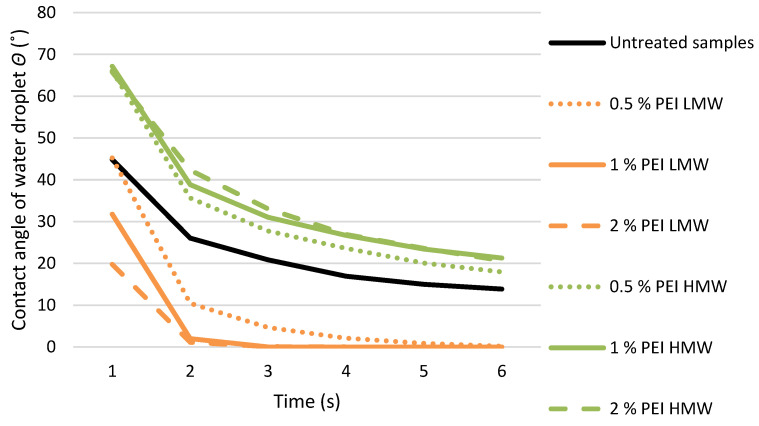
The contact angle of a water droplet on the surface of untreated samples and samples treated with PEI solution of low (LMW) and high (HMW) molecular weight of different concentrations, during the observation period from 1 to 25 s.

The results of the change in the contact angle of the water droplet over time on the surface of the untreated samples and samples treated with PEI solutions are shown in the graph (Figure 9).

In general, the value of the contact angle decreases with time, which is due to the spreading of the water droplets and the uptake of the water in the wood [42,43]. According to a previous study in which the water uptake of oak veneers treated with different polyelectrolyte solutions was investigated, the decrease in the water contact angle of the treated samples over time is to a greater extent due to the evaporation of water from the surface of the samples and to a lesser extent due to the uptake of water into the wood [13].

The water-wetting of the surface of samples treated with a polyelectrolyte solution depends on the molecular weight of the dissolved PEI polyelectrolytes. Samples treated with a PEI LMW solution showed lower contact angle values and thus better wetting with water. One of the factors determining the wetting of liquid droplets on the wood surface is the surface structure of the substrate [43,44]. After treating the wood with an aqueous solution of polyelectrolytes, the roughness of the wood surface increases, which probably influenced the reduction in the contact angle, as it is known that the roughness of the wood promotes the wetting process [45].

Higher water contact angle values were measured on the samples treated with HMW solution compared to the untreated samples, which can be explained by differences in the penetration depth and location of PEI accumulation with higher molecular mass in the wood surface layer. This finding confirms the results of previous studies that an increase in PEI molecular weight reduces the wettability of biofilms on agar substrate [46]. The higher values of the water contact angle on the surface of veneer samples of white pine (*Pinus sylvestris*) and oak (*Quercus robur*) treated with different polyelectrolyte solutions were attributed to the change in the wood surface [13].

**Figure 10:**
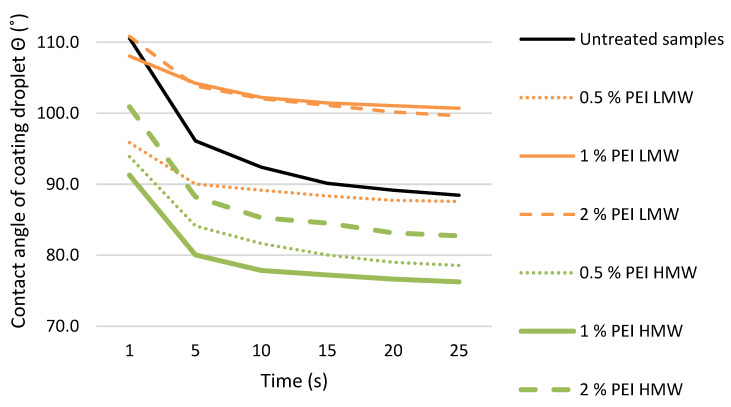
The contact angle of the WTAC droplet on the surface of untreated samples and samples treated with PEI solution of low (LMW) and high (HMW) molecular weight of different concentrations, during the observation period from 1 to 25 s.

The results of the change in the contact angle of the WTAC droplet as a function of time are shown in the graph (Figure 10).

In contrast to the results of wetting with water, wetting with WTAC had almost the opposite effect on the samples treated with PEI solutions of different molecular weights. When comparing all treated sample groups, the samples treated with 1% PEI HMW solution had the lowest values of WTAC droplet contact angle on the wood surface, which at the same time had the highest values of water droplet contact angle (both at the beginning and after 5 min of observation). Even with untreated wood, the contact angle of the coating droplet remains larger than the contact angle of the water droplet during the observation period. This result is unexpected considering that the surface tension of water (0.072 N/m) is significantly higher than the surface tension of the coating (0.02815 N/m). It is possible that the density and viscosity of the undiluted WTAC used are the reason for the high value of the contact angle of the coating droplet, as well as the fact that the evaporation rate of the water from the coating droplet is significantly slower than that of the water droplet. These results confirm the conclusion from previous studies that the wetting of the wood surface with water should not be regarded as an indicator of the quality of the wetting of the wood surface with water-based coatings [47].

The surface energy results are given in Table 3.

The samples treated with the PEI HMW solution showed a significant reduction in total surface energy compared to the untreated wood (16.7, 23.2% and 29.1% for concentrations of 0.5%, 1% and 2%, respectively). The highest values of the contact angle of the water droplets were also calculated for the same sample groups. Considering that the method for determining the surface energy is based on measuring the contact angle of liquid droplets with known surface tension, it is clear that lower values of surface energy can be explained by higher values of the contact angle of water droplets on the surface of the treated samples. In previous research [46], polyelectrolyte coating is described as a simple and efficient method to control surface energy, but our results show that this is only true for high-molecular-weight polyelectrolytes.

**Figure 11:**
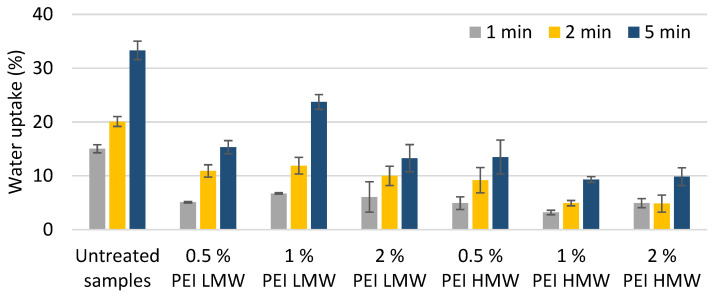
Water uptake of untreated samples and surface treated samples with PEI LMW and PEI HMW solutions of different concentrations (0.5%, 1%, and 2%).

The results of water uptake are shown in the graph (Figure 11).

All samples treated with polyelectrolyte solution showed a lower degree of water uptake compared to the untreated samples. It is expected that during sample preparation by cutting, milling and especially sanding, new fibrillar structures are opened in the surface layer of the wood, forming new pores between the fibril sections [3]. When water evaporates from the swollen wood fibers, it is possible that the newly formed pores between individual fibers close, which reduces water uptake when rewetting with water from a subsequent water-based coating. The occurrence of irreversible pore closure is associated with water evaporation during the initial drying of wood in the green moisture state (after cutting) [48] but has also been observed during the removal of water from the cell walls of wood fibers in the papermaking process [49]. In addition to a possible pore closure, the swollen wood fibers in the surface layer of the wood physically block the penetration of water into the deeper layers of the wood tissue, which certainly influences the degree of water uptake [3].

The analysis of the effect of the treatment of polyelectrolyte solutions with different molecular weights on the degree of water uptake showed that when PEI HMW is used, lower water uptake of the treated samples can be obtained than in samples treated with the same type of polyelectrolyte but with a low molecular weight. This result is consistent with the conclusions of previous studies [13], according to which the water uptake of pine veneers treated with polyelectrolytes was more pronounced with polyelectrolytes of higher molecular weight. Since high-molecular-weight polyelectrolytes can only penetrate the cell wall to a limited extent (Figure 8c), it is assumed that high-molecular-weight polyelectrolytes coat the lumens of the cell walls and form a barrier for the penetration of water molecules into the interior of the wood tissue, which is also confirmed by epifluorescence microscope images.

The results of the degree of water uptake showed no clear correlation between the concentration of the polyelectrolyte in the solution and the degree of water uptake of the samples treated with the selected solution. The lowest water uptake was observed in the samples treated with 1% PEI HMW (78.7% and 72% lower uptake of water droplets compared to untreated samples after 1 and 5 min of observation), confirming that the concentration of the polyelectrolyte solution is a key parameter for optimizing the polyelectrolyte solution for minimal water uptake on wood surfaces of certain species [13].

The treatment with PEI LMW reduced the water uptake of the beech wood samples only at the highest concentration in the solution of 2% when observing the long-term effect (after 5 min observation time). In addition, it was found that a 2% concentration of PEI solution resulted in a more stable increase in the mass of the samples in contact with water, regardless of the MW of PEI.

Reduced water uptake can be of particular importance when using wood outdoors, where the resistance of the coated surface to liquid water determines the use category of the product (stable, semi-stable or unstable end product according to EN 927-2: 2014 [50]).

### 3.4. Results of the Dry Coating Film on the Surface of Wood Treated with PEI Solution

The results of the dry film thickness WTAC on untreated and treated samples are shown in Table 4.

A one-way ANOVA showed that there was a statistically significant difference in DFT between untreated and PEI LMW and PEI HMW treated samples (F(6,203) = 23.65 at *p* < 0.5). The Games–Howell post hoc analysis revealed that the samples treated with 2 % PEI HMW had significantly higher DFT compared to the other sample groups. These results are consistent with epifluorescence microscope images showing that high-molecular-weight PEI is retained on the wood surface and forms a barrier to coating penetration (Figure 7d–f). The DFT of the samples treated with a 0.5% PEI solution with both molecular weights was slightly lower, but still significantly higher than the DFT of the untreated samples and all other groups of treated samples. These results indicate that at the low concentration, the water from the polyelectrolyte solution had a preeminent effect on the PEI, resulting in a higher DFT due to the narrowing of the penetration pathway and grain raising of the wood fibers on the surface.

### 3.5. Penetration Parameters of WTAC

Table 5 shows the basic parameters of WTAC coating penetration for untreated samples and samples treated with PEI of different MW and concentrations.

One-way ANOVA showed significant differences between the *D_max_* of WTAC between untreated samples and samples treated with PEI of different MW and concentrations (F(6,195) = 11.246 for *p* < 0.5). The post hoc Tukey test revealed that the group of samples treated with PEI HMW of all concentrations and with 2% PEI LMW had a statistically lower penetration depth compared to the untreated samples.

The groups of treated samples that had a lower maximum penetration depth also had a lower average penetration depth (F(6,195) = 14.237 at *p* < 0.5). As shown in Table 4, the untreated samples with the highest penetration depth had the lowest DFT. The lower penetration depth of the water-based coating in the treated samples can be attributed to the swelling of the cell walls of the wood and the associated narrowing of the penetration paths, which was confirmed on the SEM images (Figure 8b,c). The average penetration depth (*D_av_*) of WTAC decreased by 36% when the wood surface was treated with 1% PEI HMW. When the samples were treated with lower concentrations of PEI LMW (0.5 and 1%), the penetration depth (both *D_max_* and *D_av_*) was not statistically lower compared to the untreated samples.

With regard to the filling of the available cell lumens (parameter LF), there was no difference between untreated and polyelectrolyte solution-treated samples (F (6,163) = 1.007 at *p* = 0.423). The statistical homogeneity of the LF values confirms that the possibilities of penetration of the coating into the wood depth were uniform in all sample groups so the influence of structural elements on the penetration depth can be excluded.

### 3.6. The Adhesion Strength of WTAC Coating

Table 6 shows the results of the adhesion strength of the WTAC in the untreated and treated samples.

A one-way ANOVA with Tukey’s HSD had shown that treatment of the samples with 1% PEI LMW and 0.5% PEI HMW resulted in significantly lower adhesion strength of WTAC to the wood surface compared to untreated samples (F(6,193) = 4.714 for *p* < 0.5). All other groups of treated samples showed lower values for the adhesion strength of WTAC, but the difference was not statistically significant. The SEM images showed that the treatment with polyelectrolyte solutions leads to a filling of the cell walls, which can lead to a weakening of the mechanical properties of the wood in the surface layer [51], as the number of microfibrils per unit area decreases. Since the adhesion of the coating depends on the mechanical properties of the substrate [52], the filling of the cell walls can reduce the adhesion of the coating and the wood. On the other hand, treatment with polyelectrolyte solutions led to an increase in roughness in the surface layer of the wood, so that the contact zone between the coating and the wood is larger. Tests have shown that mechanical bonding is one of the most important adhesion mechanisms of water-based coatings [53]. It is possible that the increase in roughness and thus the increase in the contact zone between the coating and the wood in the treated samples has reduced the losses in the adhesive bonds that are to be expected due to the decrease in the mechanical properties of the cells in the surface layer of the wood.

### 3.7. The Surface Roughness of the Wood Coated Surface

Table 7 shows the roughness parameters *R_a_*, *R_z_* and *R_t_* of the wood-coated surface.

All samples treated with PEI solutions of different MW and concentrations show an improvement in surface quality in the form of a reduction in surface roughness. The best result in terms of the roughness of the coated surface was shown by the samples treated with a 1% PE HMW solution, where the roughness of the coated surface was reduced by 44.5, 40.8, and 45.0% for the parameters *R_a_*, *R_z_*, and *R_t_*, respectively (compared to untreated samples). A statistically significant difference was found between the untreated samples and the samples treated with PEI LMW solutions and PEI HMW solutions with respect to the parameters *R_a_* (F (6,203) = 140.40 for *p* < 0.5), *R_z_* (F (6,203) = 80.96 for *p* < 0.5) and the parameter *R_t_* (F (6,203) = 55.10 for *p* < 0.5).

Within the treated samples, the lowest reduction in surface roughness in terms of *R_a_* and *R_z_* was observed in the samples treated with 0.5% PEI LMW, while the highest value for *R_t_* was obtained in the samples treated with 0.5% PEI of both MW, with no statistically significant difference between these two groups.

## 4. Conclusions

The aim of this study was to investigate the influence of treating the wood with weak cationic polyelectrolyte, PEI, on the adhesion of the water-based transparent acrylic coating to the wood surface. The conditions for the deposition of the polyelectrolyte were varied by using PEI with two different molecular weights—low-molecular-weight PEI (PEI LMW) and high-molecular-weight PEI (PEI HMW).

The results of this study suggest that by treating the surface of beech wood prior to coating, water uptake can be reduced by 72% when the surface is treated with a 1% solution of PEI HMW (compared to untreated samples after 5 min of applying water droplets to the surface). This result is illustrated by the significant reduction in surface energy of the treated surface by 23.2% compared to the untreated surface. Treatment with a 1% PEI HMW solution resulted in the lowest WTAC wetting angles and the highest water droplet wetting angles compared to other combinations of molecular weight and concentration of PEI solution.

PEI LMW solutions had a greater effect on reducing sample surface water uptake only at the highest solution concentration of 2%. The reduction in water uptake of the treated wood samples had a positive effect on the roughness of the coated surface, which is of particular importance when water-based coatings are used for the surface treatment of wood.

It was found that the molecular weight has an influence on the penetration depth of the PEI solution at all concentrations (0.5, 1 and 2%), so that PEI HMW should be used if the polyelectrolyte network is to be retained in the outer layer. If, on the other hand, deeper penetration of PEI into the interior of the wood is required, PEI LMW solutions should be used. These results were confirmed by epifluorescence and SEM microscopy.

Treatment with a PEI solution of different concentrations and MW did not reduce the adhesion strength of WTAC, which allows the introduction of this process into industrial production without compromising the protective properties of the coated surface. This exception was observed in the samples treated with 1% PEI LMW and 0.5% PEI HMW. In industrial practice, the surface treatment of wood with 1% PEI HMW can be used before the wood is finished with water-based coatings to improve the surface properties in terms of roughness. This is particularly important for small and medium-sized businesses that do not have intensive and fast-working dryers, because a straightforward surface treatment, can improve the quality of the finished product. Including the 1% PEI HMWsurface treatment in the wood surface finishing would require industrial application techniques (e.g., spraying), which would change the viscosity of the coating and possibly its penetration depth. The higher penetration depth expected due to the lower viscosity of the coating could even increase the adhesion strength of the water-based coating to the wood surface. In the meantime, according to our findings, the 1% PEI HMW treatment of the wood maintains the ability of the water-based coating to sufficiently adhere to the treated surfaces.

## Figures and Tables

**Table 1:** Specification of used polyelectrolytes.

Polyelectrolyte Type	Manufacturer	Molecular Weight, g/mol	pH	Abbreviation	Concentration, %
Polyethyleneimine (PEI)	Sigma-Aldrich, St. Lous, MO, USA	1800	12.0	PEI LMW	0.5 1 2
	MP Biomedicals, Illkirch, France	50,000–100,000	10.5–11.0	PEI HMW	0.5 1 2

**Table 2:** Properties of liquid WTAC.

Density, g/mL (EN ISO 2811-1: 2016) [22]	Solids Content, % (EN ISO 3251: 2019) [23]	Flow Time, s (EN ISO 2431: 2012) [24]	Apparent Viscosity, mPa × s (EN ISO 2555: 2018) [25]	pH Value	Surface Tension, N/m (ISO 304: 1985) [26]
1.024	31.3	55.5	3460	7.34	0.02815

**Table 3:** Results of total surface energy (γ) of untreated samples and samples treated with PEI solutions of different MW and concentration.

Group of Samples		Concentration, %	Surface Energy (γ), N/m
Untreated samples		/	0.0587
Treated samples	PEI LMW	0.5	0.0576
		1	0.0575
		2	0.0550
	PEI HMW	0.5	0.0489
		1	0.0451
		2	0.0416

**Table 4:** Dry film thickness of WTAC of untreated samples and samples treated with PEI of different MW and concentration.

Dry Film Thickness (DFT) of WTAC *, µm						
Untreated samples	Treated samples					
	PEI LMW			PEI HMW		
	Concentration, %			Concentration, %		
57.80 (5.27)	0.5	1	2	0.5	1	2
	64.57 (4.17)	58.07 (6.05)	55.63 (7.50)	61.80 (4.50)	59.73 (3.11)	69.83 (6.35)

* Values in parenthesis are standard deviations.

**Table 5:** Penetration parameters of WTAC untreated and samples treated with PEI solution of different concentrations: Maximum penetration depth (*D_max_*); average penetration depth (*D_av_*) and lumen filling (*LF*).

Samples		Concentration, %	Penetration Parameters of WTAC		
			*D_max_* *, µm	*D_av_* *, µm	*LF* *, %
Untreated samples		/	84.18 (19.40)	43.31 (11.67)	37.6 (18.8)
Treated samples	PEI LMW	0.5	80.05 (18.17)	48.33 (13.26)	29.5 (16.4)
		1	68.65 (21.60)	37.55 (11.17)	37.8 (19.6)
		2	56.95 (16.71)	32.20 (8.36)	55.3 (21.3)
	PEI HMW	0.5	57.56 (11.59)	33.21 (5.86)	40.3 (22.1)
		1	66.12 (19.27)	30.13 (8.37)	32.3 (16.5)
		2	62.35 (13.44)	35.46 (6.53)	47.7 (15.8)

* Values in parenthesis are standard deviations.

**Table 6:** The adhesion strength of WTAC on the surface of untreated and samples treated with PEI of different MW and concentration.

Samples		Concentration, %	Adhesion Strength *, MPa
Untreated samples		/	3.54 (0.58)
Treated samples	PEI LMW	0.5	3.44 (0.59)
		1	3.10 (0.43)
		2	3.28 (0.47)
	PEI HMW	0.5	3.15 (0.38)
		1	3.55 (0.51)
		2	3.50 (0.48)

* Values in parenthesis are standard deviations.

**Table 7:** The surface roughness parameters *R_a_*, *R_z_* and *R_t_* of the coated surface of untreated samples and samples treated with PEI of different MW and concentration.

Group of Samples		Concentration, %	The Roughness of the Varnished Wood Surface *, µm		
			*R_a_*	*R_z_*	*R_t_*
Untreated samples		/	6.659 (0.752)	38.84 (5.42)	54.20 (9.66)
Treated samples	PEI LMW	0.5	5.508 (0.653)	32.91 (4.74)	43.44 (5.98)
		1	3.949 (0.418)	24.55 (2.21)	32.41 (4.22)
		2	4.253 (0.546)	26.14 (2.98)	34.25 (3.51)
	PEI HMW	0.5	4.106 (0.537)	26.71 (3.50)	38.80 (6.73)
		1	3.318 (0.287)	21.14 (2.90)	30.93 (4.02)
		2	3.997 (0.344)	24.35 (2.87)	33.86 (6.04)

* Values in parenthesis are standard deviations.

## Data Availability

The original contributions presented in the study are included in the article, further inquiries can be directed to the corresponding authors.
